# A zoonotic *Streptococcus equi* subsp. *zooepidemicus* strain survives in activated blood neutrophils of pigs through SzM expression

**DOI:** 10.1128/iai.00113-26

**Published:** 2026-07-15

**Authors:** Sophie Öhlmann, Christine Weiße, Andreas Nerlich, Tanja Wurzer, Yannick Max Fornoville, Anna Majcher, Thomas Grochow, Simone A. Fietz, Reiner Ulrich, René Bergmann, Christoph Georg Baums

**Affiliations:** 1 Institute of Bacteriology and Mycology, Centre for Infectious Diseases, Faculty of Veterinary Medicine, Leipzig University 9180, Leipzig, Germany; 2 School of Veterinary Medicine, Veterinary Centre for Resistance Research (TZR), Freie Universität Berlin 9166, Berlin, Germany; 3 Institute of Veterinary Pathology, Faculty of Veterinary Medicine, Leipzig University 9180, Leipzig, Germany; 4 Institute of Veterinary Anatomy, Histology and Embryology, Faculty of Veterinary Medicine, Leipzig University 9180, Leipzig, Germany; Tsinghua University, Beijing, China

**Keywords:** *Streptococcus equi *subsp. *zooepidemicus*, SzM, M-Protein, intracellular survival, neutrophils, oxidative burst, phagocytosis

## Abstract

*Streptococcus equi* subsp. *zooepidemicus* (SEZ) causes diseases in various animals and humans. In pigs, it is an emerging pathogen that causes septicemia and other severe pathologies. SEZ expresses the M-like protein SzM, which recruits host proteins such as fibrinogen to the bacterial surface. The objective of this study was to investigate the survival mechanism of a zoonotic SEZ strain in porcine blood. The zoonotic SEZ C33 strain proliferated in porcine blood of 10-week-old piglets *in vitro* despite comparably high levels of IgM binding to the bacterial surface. Comparison with the isogenic *szm* deletion mutant showed that SzM_C33 expression is crucial for porcine blood survival. Flow cytometry analysis revealed that SEZ C33 was associated with porcine blood granulocytes, exhibiting a prominent generation of reactive oxygen species. Cytochalasin D treatment significantly inhibited this association. Furthermore, we found viable intracellular SEZ C33 in a penicillin/streptomycin protection assay in porcine blood and purified porcine granulocytes, in agreement with the microscopic detection of streptococci in neutrophils. Experimental infection with SEZ C33 confirmed the virulence of this strain in pigs, including one case of fibrino-suppurative meningoencephalitis and tarsitis. In conclusion, SEZ C33, originally isolated from a diseased human, demonstrated SzM-mediated intracellular survival in porcine blood granulocytes *in vitro* and is most likely involved in the pathogenesis of diseases such as meningitis and arthritis *in vivo*.

## INTRODUCTION

*S. equi* subsp. *zooepidemicus* (SEZ) can cause diseases in very different species, such as horses (1–4), dogs (5–7), ruminants (8–11), pigs (12–14), poultry (15), monkeys (16), and humans (17). In humans, SEZ may cause bacteremia, meningitis, arthritis, and endocarditis. Many of the reported cases in humans have been related to the consumption of unpasteurized milk products or contact with horses (17–19).

SEZ has caused outbreaks of severe disease with high mortality in pigs in China (20, 21), Indonesia (16), and North America (13, 14). Lethargy, fever, reluctance to rise, and cutaneous erythema are described signs of SEZ septicemia in pigs, which might rapidly progress toward death in association with the detection of SEZ in multiple inner organs (12, 14, 22). Piglets and sows may also be affected. Clinically inapparent carriers most likely play an important role in the epidemiology of SEZ outbreaks. However, the factors that lead to disease outbreaks are not well understood. SEZ isolates from diseased pigs in a recent outbreak in North America were similar to the Chinese outbreak strain (13). Outbreak isolates have been shown to be more virulent in experimental infections than isolates from other animals; however, loss-of-function experiments to identify crucial virulence factors have not been conducted in pigs.

SEZ expresses at least one M-like protein such as SzP or SzM (23, 24). Similar to the M protein of *S. pyogenes,* these M-like proteins are most likely also dimeric coiled-coil structures, are surface-associated, and recruit host proteins to the bacterial surface. We investigated the function of two different SzM proteins (SzM_C2 and SzM_C33) in previous studies, including loss-of-function experiments with strains SEZ C2 and SEZ C33 (24, 25), respectively. Both proteins bind to the Fc region of IgG (24). Furthermore, SzM_C33 is necessary in strain SEZ C33 for the recruiting of equine and human fibrinogen to the bacterial surface (24). The binding of human fibrinogen is less pronounced in SEZ C2 in contrast to the binding of equine fibrinogen (24). Non-immune binding of C1q to the bacterial surface via SzM_C2 is a distinct characteristic of SEZ C2 and is involved in complement evasion (25). In this study, we comparatively investigated the survival of SEZ C2 and C33, as well as their isogenic *szm* deletion mutants, in porcine blood. After detecting a distinct phenotype of the zoonotic strain SEZ C33, we further characterized the SzM_C33-mediated interaction with porcine neutrophils.

## MATERIALS AND METHODS

### Bacterial strains and growth conditions

SEZ strain C33 was originally isolated from the blood of a human patient with pneumonia, pericarditis, and septicemia in Germany in 2011. The SEZ strain C2 was isolated from the bronchoalveolar lavage of a horse in Germany in 2015. The inframe deletion of the *szm* gene was conducted within our previous study to generate the isogenic mutants C33Δ*szm* and C2Δ*szm* (24).

SEZ was grown at 37°C without agitation in Todd-Hewitt broth supplemented with 0.5% yeast extract (THY).

*E. coli* strain XL1 blue carrying pQE30_SzM_C2 or pQE30_SzM_C33 (24) was cultured in Luria Bertani (LB) broth or on LB agar containing ampicillin at 100 µg/mL at 37°C.

### Scanning transmission electron microscopy

Samples were fixed according to the lysine acetate–ruthenium red fixation protocol to preserve the polysaccharide capsule (26). Briefly, bacterial cells were prefixed for 20 min in 2% paraformaldehyde and 2.5% glutaraldehyde in 0.1 M cacodylate buffer containing 0.075% ruthenium red and 0.075 M lysine acetate. Samples were subsequently postfixed for 3 h in the same fixative lacking lysine acetate. Thereafter, samples were embedded in 1.5% low-melting-point agarose and postfixed for 1 h in 1% osmium tetroxide. Following dehydration through a graded ethanol series, specimens were stained for 1 h with 0.5% uranyl acetate and embedded in epoxy resin. Ultrathin sections (50 nm) were prepared and counterstained with 2% uranyl acetate and Reynolds’ lead citrate. Images were acquired using a Zeiss EVO LS 15 electron microscope equipped with a STEM detector (Carl Zeiss Microscopy GmbH, Oberkochen, Germany). Capsule thickness was determined as the mean of the minimum and maximum capsule thickness measured for each bacterium. A minimum of 50 bacterial cells were analyzed per sample.

### Whole-genome sequencing

DNA of SEZ strains C2 and C33 was isolated using DNeasy Blood and Tissue Kit for DNA Isolation (Qiagen, 69506) according to manufacturer’s instructions and submitted to Illumina NovaSeq 6000 full genome sequencing. The sequencing data were analyzed on Galaxy platform (27) using the following tools: Falco for quality control (28), Trimmomatic to trim paired Ilumina-reads (29) with the following parameters: SLIDINGWINDOW 4:20, MINLEN 50, LEADING 3, and TRAILING 3, after an initial ILLUMINACLIP step, clipping the nextera paired-end adapter sequences with the standard values of the tool. Furthermore, we used Unicycler to assemble the trimmed reads (30), FastANI to compare both assemblies (31), Prokka for annotation with standard parameters (32, 33), Roary to compare annotated sequences (34), and MLST (35). To get information for assembly submission, we used Samtools coverage (36, 37) on the Unicycler-Assembly and mapped the trimmed reads against the assembled genomes with BWA-MEM2 (38–40). Scaffolds assembled with Unicycler v0.5.1 were submitted to the NCBI Database. The whole-genome shotgun sequencing project has been deposited at DDBJ/ENA/GenBank under accession PRJNA1462165 with number JBYAWY000000000 for SEZ C33 and number JBYAWZ000000000 for SEZ C2.

Alignment of SzM_C2 and C33 was performed using Clustal Omega web service (program clustalo v1.2.4) (41) provided by the EMBL-EBI (42) with default parameters using FASTA data of SzM_C2 partial (AZW07476.1) and SzM_C33 partial (AZW07490.1). The used sequences represent the mature proteins starting at the signal sequence and containing the LPSTG motif.

### Bactericidal assay in whole blood

To determine the survival of SEZ in heparinized porcine blood, assays were conducted as previously described (24) using an infection dose of 2 × 10^6^ CFU/mL. The survival factor was calculated by dividing colony-forming units (CFU) after 120 min incubation by the CFU directly after inoculation at time zero.

### ELISA

As SzM can lead to nonspecific binding of antibodies, IgM and IgG levels were detected using plates coated with the respective inactivated SEZ *szm* deletion mutant. Coating of the plates and subsequent ELISA were performed as previously described (43). A reference serum containing opsonizing antibodies against different streptococci was defined as containing 100 ELISA units of both IgG and IgM, and the ELISA units were calculated relative to this reference.

### Western blot analysis

Plasma absorption assays with western blot analysis were performed as described previously (24), except for the use of porcine plasma instead of human or equine plasma. After blotting, the nitrocellulose membrane was incubated with rabbit anti-pig fibrinogen (ABIN491121, antibodies-online, 1:5,000) and goat anti-rabbit IgG HRP (111-035-008, Dianova, 1:3,000) antibodies.

### Detection of granulocytes in flow cytometry

Fresh whole blood was collected from two 7-week-old pigs into lithium heparin-coated tubes. An aliquot of 1.5 mL per pig was used for the experiment. Erythrocytes were lysed by repeated hypotonic lysis: 8 mL of 0.2% sodium chloride was added to each tube, followed by the same volume of 1.6% sodium chloride after 30 s to stop the lysis reaction. The tubes were centrifuged at 400 × *g* for 6 min, and the supernatants were discarded. After counting using trypan blue (Sigma-Aldrich, T8154), the cells were distributed into a 96-well plate at 1 × 10^6^ cells per well in 100 µL. The following steps were carried out on ice or at 4°C. First, a blocking step was performed with 20 µL/well of blocking buffer (30% heat-inactivated porcine serum in FACS buffer [PBS containing 3% fetal bovine serum [FBS superior, Sigma-Aldrich, S0615] and 0.1% sodium azide]) for 5 min. The cells were then stained with the following antibodies for 30 min, 20 µL per well: (i) Mouse anti-pig CD14 MIL2 (IgG2b, purified; from Bio-Rad MCA1218GA; 1:20 in blocking buffer) or purified Mouse IgG2b isotype control (BioLegend, 400301, 1:20 in blocking buffer) (for FMO_CD14); (ii) APC anti-mouse IgG2b (rat IgG1; BioLegend RMG2b-1; 1:20 in FACS-buffer); (iii) Mouse anti-pig SWC8 MIL3 (IgM; Bio-Rad MCA1219; 1:8 in FACS-buffer) or FACS-buffer (FMO_SWC8); (iv) PE anti-mouse IgM (rat IgG2a; Miltenyi 130-095-908; 1:1 in FACS-buffer). Between each staining step, cells were washed twice with FACS-Buffer. Afterward, the cells were washed twice with PBS, resuspended in 100 µL of 2% (wt/vol) paraformaldehyde, and fixed for 30 min. After washing once with FACS buffer, cells were analyzed using a BD LSRFortessa flow cytometer.

Granulocytes were gated using forward and side scatter; single cells were identified, and the percentage of CD14+ SWC8+ cells in the granulocyte gate was determined (Fig. S3).

### Detection of SEZ-induced oxidative burst in granulocytes

Reconstitution of blood and detection of phagocytosis, in combination with an oxidative burst, was performed as previously described (44). Briefly, SEZ C2 or C33, prestained with CellTrace FarRed (Invitrogen, C34564) as described previously (45), were preincubated in the sera of 6-week-old piglets and added to porcine blood cells to reach a final concentration of 2 × 10^6^ CFU/mL in the reconstituted blood. Staining of the SEZ was verified by measuring bacteria with BD LSRFortessa (Table S1A). Samples with phosphate-buffered saline (PBS) and PMA (Phorbol-12-myristat-13-acetat; Sigma-Aldrich, 0.18 µg/mL,) were included as negative and positive controls, respectively. After incubating the samples at 37°C for 15 min, dihydrorhodamine 123 (Sigma-Aldrich, D1054, DHR123; 5 μg/mL) was added. DHR123-treated samples were incubated for 10 min at 37°C, and DHR123 was oxidized to the fluorescent marker rhodamine 123 (Rho123) in the presence of reactive oxygen species. Erythrocytes were lysed twice with lysis buffer (0.155 M ammonium chloride, 10 mM potassium bicarbonate, 0.1 mM disodium EDTA, pH 7.2). Leukocytes were washed twice with PBS, fixed with 2% (wt/vol) paraformaldehyde, and measured immediately by flow cytometry (BD FACSCalibur). Data analysis was conducted using gating granulocytes (FlowJo).

### Fluorescence analysis with wheat germ agglutinin-Alexa-Fluor 488

Porcine blood was infected with SEZ strain C33 (9 x 10^6^ CFU/mL) for 60 min at 37°C under constant rotation. Red blood cells were lysed with distilled water for 30 s, followed by the addition of 10× PBS (1:10) to stop the lysis process and centrifugation at 700 × *g* for 5 min. The resulting cell pellet was fixed in 4% buffered formaldehyde solution (AppliChem, A3697) for 10 min at room temperature, centrifuged, and resuspended in PBS. The fixed cell suspensions were permeabilized with PBS containing 0.05% saponin (PBS-S) and subsequently blocked in PBS-S containing 1% (wt/vol) BSA and 5% (vol/vol) FCS. The cells were then incubated in a solution of Hoechst 33342 (ThermoFisher Scientific, Cat. No. H1399; 1:1,000 dilution) and wheat germ agglutinin (WGA) Alexa Fluor 488 (ThermoFisher Scientific, Cat. No. W11261, 1:500 dilution) in PBS-S for 20 min. All washing steps were performed in PBS-S for 5 min after collecting the cells by centrifugation at 700 × *g*. Stained cells were applied to Poly L-Lysin-coated No. 1.5H coverslips and mounted on glass slides using ProLong Glass (ThermoFisher Scientific, Cat. No. P36984).

Confocal microscopy was performed using an inverted Leica Stellaris 8 FALCON system, equipped with a 405 nm laser, a white light laser (WLL, 440–790 nm), acousto-optical beam splitter, Power HyD detectors, and controlled by LAS X (version 4.8.1.29271). Images were acquired with an HC PL APO CS 63×/1.40 oil immersion objective, an image pixel size of 36 nm, and a z-step size of 0.3 µm. DAPI was excited using a 405 nm laser at 1.5% intensity, while Alexa Fluor 488 was excited at 499 nm using the WLL set to 2.0% intensity. Fluorescence emission was collected between 430 and 504 nm for DAPI and between 504 and 663 nm for Alexa Fluor 488 using HyD S detectors in analog mode (gain 2.5), and the pinhole was set to one airy unit by applying unidirectional line sequential acquisition mode at a scan speed of 600 Hz. Images were further processed using LAS X LIGHTNING with “Mounting Medium” set to ProLong Glass (RI = 1.52), and all other settings were left as default. Brightness and contrast were linearly adjusted in ImageJ/FIJI (46) for display purposes, and images were displayed as maximum intensity projection (*xy*) and orthogonal views (*xz* and *yz* along the indicated lines in the figure).

### Detection of intracellular SEZ by flow cytometry

To determine the number of granulocytes containing intracellular SEZ, trypan blue (Sigma-Aldrich, T8154) was used as a quenching agent in the flow cytometry analysis as previously described (47). SEZ was labeled with the green fluorescent dye CellTrace CFSE (Invitrogen, C34570), and the blood of nine 7-week-old piglets was incubated with 2 × 10^6^ CFU/mL SEZ*CFSE for 30 min followed by erythrocyte lysis as described above for oxidative burst experiments. Labeling efficiency was analyzed using the flow cytometer BD LSRFortessa. To verify the intracellular location of SEZ in porcine blood granulocytes by quenching the CFSE signal of SEZ located at the outside of the cell, 1.2 mg/mL trypan blue was added just before measuring the samples using BD LSRFortessa, as described (48).

### Isolation of porcine granulocytes

Freshly collected heparinized porcine blood (permit number TV vG 01/23) was obtained from animals aged 4–9 weeks. Isolation of granulocytes was performed as described (49) with slight variations, as PBS was used instead of 0.87% NaCl, and blood was layered on Pancoll human (PAN Biotech, P04-60500). The gradient was centrifuged at 800 × *g*. After erythrocyte lysis, granulocytes were maintained in RPMI 1640 (Roswell Park Memorial Institute medium, Gibco, 21875-034) supplemented with 10% fetal bovine serum (FBS superior, Sigma-Aldrich, S0615) overnight at room temperature in the dark, and viability was assessed using trypan blue.

### Penicillin/streptomycin protection assay

To investigate whether internalized SEZ survived intracellularly, we used a penicillin/streptomycin protection assay. Blood was infected with 2 × 10^6^ CFU/mL, or purified granulocytes were infected with an MOI of 1:2 of the respective SEZ strains (5 × 10^6^ CFU to 1 × 10^7^ granulocytes). After 30 min of incubation at 37°C, 60 µg/mL Penicillin G sodium salt (Carl ROTH, HP48.2) and 100 µg/mL streptomycin sulfate (Carl ROTH, HP66.3) were added to an aliquot of each sample, and all samples were kept at 37°C under constant rotation for further 60 min. Samples were then centrifuged at 500 × *g* for 10 min at 4°C. The supernatant was removed and plated in serial dilutions to verify the absence of growth due to the bactericidal activity of the penicillin/streptomycin treatment. The cells were washed three times with PBS and subsequently lysed with distilled water for 5 min, followed by plating of serial dilutions.

### Growth and killing in the presence of granulocytes

The interaction with porcine granulocytes was investigated in an opsonophagocytosis assay including 20% (vol/vol) porcine serum, as described previously (45), but in the absence of specific antibodies as serum of colostrum-deprived piglets (CDS) was used. Briefly, 1 × 10^7^ cells/mL were suspended in RPMI 1640 containing 20% CDS and infected with 5 × 10^6^ CFU/mL of the respective SEZ strains to obtain a multiplicity of infection of 1:2. After 30 min, aliquots were taken and treated with 60 µg/mL penicillin and 100 µg/mL streptomycin for 60 min, followed by washing and lysis of the cells as described above for the penicillin/streptomycin protection assay. Serial dilutions of all samples were plated at different time points to investigate growth and killing of SEZ under these conditions. The influence of granulocytes on the growth behavior of SEZ was verified by incubation of the bacteria in RPMI with 20% CDS without granulocytes.

### Experimental infection of piglets

Six landrace piglets were infected intravenously with 1.5 × 10^7^ CFU of SEZ strain C33 grown in THY supplemented with 10% porcine serum (Sigma-Aldrich) to an OD_600nm_ of 0.9. After infection, piglets were monitored every 8 h, including scoring of clinical signs, such as increased body temperature and behavior (Tables S3 and S4). In case of acute clinical signs such as polyarthritis, central nervous system dysfunction (e.g., convulsions), or a cumulative clinical score above 25, euthanasia was performed for animal welfare reasons. All piglets underwent necropsy and histopathological and bacteriological screening, as described previously (50), with the addition of the kidney. The bacteriological screening included analysis of β-hemolytic streptococci in MALDI-TOF MA, *szm*-specific PCR, and sequencing of the resulting *szm* amplification, as previously described (24). The animal experiment was approved by Landesdirektion Sachsen (Permit No. TVV 44/23), which includes approval by the registered ethics committee for animal experiments.

### Statistical analysis

Statistical analyses were performed using Prism software version 10 (GraphPad, San Diego, CA, United States). Differences between multiple groups were determined using the Friedman test followed by Dunn’s multiple comparison test (non-parametric data) or RM one-way ANOVA and Šídák’s or Tukey’s multiple comparison test (normally distributed data). Normal distribution was tested using the Shapiro-Wilk test. All figures represent the mean ± SD. Significant differences are indicated by asterisks. The probabilities were considered as follows: *P* < 0.05 *, *P* < 0.01 **, *P* < 0.001 ***.

## RESULTS

### Comparison of equine SEZ strain C2 and human SEZ strain C33

In our previous work, we comparatively investigated SzM_C2 and SzM_C33 with respect to their binding to fibrinogen, IgG, and complement factor C1q (24, 25) and showed that SzM_C33 clusters with zoonotic strains distinct from SzM_C2. Despite shared functional properties, SzM proteins from different isolates exhibit pronounced heterogeneity. This is also evident for the two SEZ strains investigated here, as illustrated by the alignment of the mature protein sequences (Fig. S1). Despite the greater length of SzM_C2 compared to SzM_C33, the proteins share several similar but not identical regions within the N-terminus and central domains, whereas the C-terminal region, including the LPSTG anchor motif, is conserved. Overall sequence identity between the two proteins is 36.62%.

We further sought to elucidate the genomic background of the two SEZ isolates using data generated by Illumina NovaSeq 6000 sequencing. Comparative genomic analysis with the Roary tool revealed that the two isolates share 1,525 genes and differ in 735 of 2,260 totally identified genes. The average nucleotide identity (ANI) is 97.22%. The equine SEZ strain C2 belongs to sequence type (ST) 26, whereas C33 is classified as ST212 (Table S1). Notably, both strains harbor the gene encoding SzP, a second M-like protein described in SEZ.

**Fig 1:**
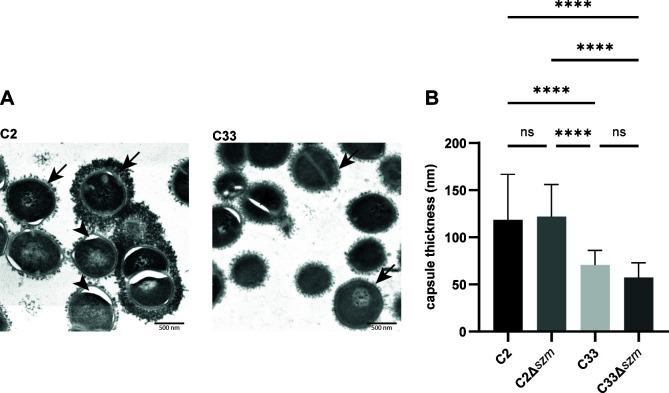
Distinct capsule morphologies of SEZ strains C2 and C33. Scanning transmission electron microscopy (STEM) images of strain C2 and strain C33 (A). Arrows indicate the glycoprotein capsule of SEZ. Arrowheads indicate shrinkage artifacts. Brightness and contrast were adjusted using Adobe Photoshop for visualization purposes only. (B) Comparison of capsule thickness between the two strains and their mutants. For each bacterial cell, capsule thickness was determined by measuring the thinnest and thickest regions of the capsule and calculating the mean of these two measurements. At least 50 bacterial cells were analyzed per sample. Data are presented as mean ± standard deviation (SD). Statistical analysis was performed using one-way ANOVA followed by Tukey’s multiple-comparison test. Significant differences are indicated (Not significant [ns] P ≥ 0.05, **** *P* < 0.0001).

When cultivated on blood agar plates, strain C2 forms larger, more mucoid colonies than C33, suggesting increased transcription of the HasABC operon and consequently higher expression of the hyaluronic acid capsule (Fig. S2). Indeed, STEM analysis revealed a significantly increased capsule thickness for C2 and C2∆*szm* compared to C33 or C33∆*szm* (C2: 118.4 nm ± 48.5 nm SD and C2Δ*szm*: 122 nm ± 34.1 nm SD vs C33: 70.7 nm ± 15.3 nm SD and C33Δ*szm*: 57.4 nm ± 15.6 nm SD) with a high diversity of capsule thickness between individual bacteria in SEZ C2 (Fig. 1). Of note, capsule thickness was not different between wild-type (WT) and the respective *szm* deletion mutants of C2 or C33.

### SzM_C33 mediates survival of SEZ C33 in blood of weaning piglets despite high levels of IgM binding to the surface of SEZ

**Fig 2:**
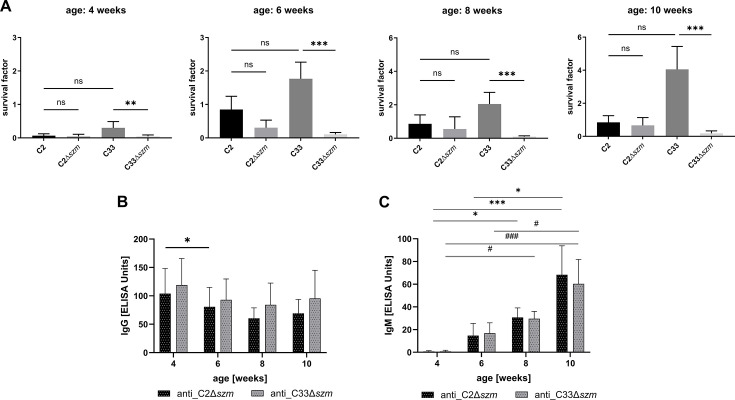
The isogenic *szm* deletion mutant of *S. equi zooepidemicus* strain C33 is significantly attenuated in survival in blood drawn from 4- to 10-week-old piglets (**A**). These piglets carry IgG recognizing SEZ independent of SzM (**B**) and are showing a prominent increase of IgM binding to SEZ between 4 and 10 weeks of age (**C**). (A) Blood was collected every 2 weeks from the same weaning piglets (*n* = 6) and inoculated with SEZ strain C2 and C33 or the indicated isogenic *szm* deletion mutants. The survival factor is defined as the ratio of CFU at 120 min to CFU at time point zero. (**B and C**) Levels of IgG and IgM were determined in ELISA using immobilized inactivated *szm* deletion mutants of SEZ as antigens because WT strains show SzM-mediated Fc-binding of IgG. Bars and error bars represent mean values and standard deviations, respectively. Statistical analysis was performed using Friedman and Dunn’s multiple comparison test (not normally distributed data in **A and C**) or RM one-way ANOVA and Tukey’s multiple comparison test (**B**). Significant differences are indicated (Not significant [ns] *P* ≥ 0.05, * or # *P* < 0.05, **P < 0.01, *** or ### *P* < 0.001).

Bacteraemia is a hallmark of invasive streptococcal infections. The expression of the SzM protein mediates the survival of SEZ strains C2 and C33 in human blood. Furthermore, SEZ strain C33 proliferates in equine blood, whereas the isogenic mutant C33Δ*szm* is efficiently killed (24). In this study, we investigated the survival of these two (WT) strains in comparison with their isogenic *szm* mutants in blood drawn from the same six piglets at the ages of 4, 6, 8, and 10 weeks (Fig. 2). SzM expression mediated the survival of SEZ C33 in the blood drawn from these piglets at all investigated time points. The older the pigs got, the survival factor of SEZ strain C33 in porcine blood increased resulting in prominent proliferation in the blood of 10-week-old piglets (Fig. 2). In contrast, the mean survival factor of SEZ strain C2 was below 1 at all investigated time points. Furthermore, the isogenic mutant C2Δ*szm* was only slightly attenuated compared with its WT strain in blood drawn at an age of 6 weeks.

Piglets receive maternal IgG against various pathogens via colostrum uptake during the first 24 h. We figured that the levels of IgG binding to SEZ in serum might be different at the time points investigated in bactericidal assays due to the decline of maternal IgG. As SzM binds to the Fc part of IgG (24), we used both inactivated *szm* deletion mutants as antigens in ELISA. As shown in Fig. 2B, the levels of IgG binding to C2Δ*szm* and C33Δ*szm* decreased between 4 and 8 weeks of age. This suggests that SEZ-reactive IgG antibodies are of maternal origin at 4 weeks of age. We have recently shown that porcine IgM plays an important role in bactericidal immunity against *Streptococcus suis* and that IgM binding to this invasive pathogen is prominently increased after weaning in association with activation of B1-like cells (51–53). In this study, we investigated the course of IgM binding to two *szm* SEZ deletion mutants. As depicted in Fig. 2C, the levels of IgM binding to C2Δ*szm* and C33Δ*szm* significantly increased between 4 and 10 weeks of age.

In summary, SEZ strain C33 demonstrated SzM-mediated survival in the blood of piglets of different ages. This strain proliferates also in the blood of older piglets in the presence of high levels of SEZ C33-binding IgM.

### SzM_C33 mediates the association of streptococci with porcine granulocytes and their activation

**Fig 3:**
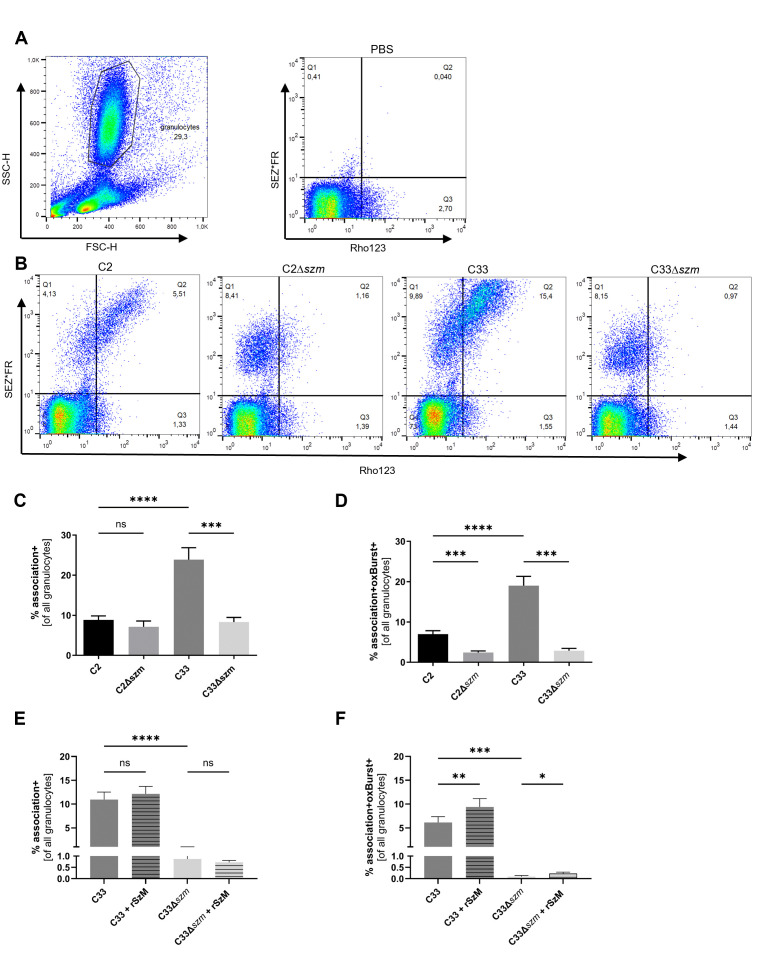
SzM_C33-dependent association of SEZ with porcine blood granulocytes and oxidative burst in these granulocytes. Sera collected from 6-week-old growing piglets (*n* = 6) were used to reconstitute porcine blood, which was incubated with 2 × 10^6^ CFU/mL of Cell Trace FarRed-labeled bacteria before adding dihydrorhodamine (DHR) 123. DHR123 enters the cells and is oxidized to fluorescent Rhodamin123 (Rho123) in the presence of reactive oxygen species. Frequency of granulocytes showing oxidative burst and phagocytosis activity induced by SEZ was measured by flow cytometry (BD FACSCalibur) and gating on SSC^high^ granulocytes. (**A**) Gating strategy for porcine blood granulocytes (SSC^high^, left) in blood incubated with PBS and DHR123 as negative control (right). (**B**) Example of flow cytometry plots of one pig. (**C and E**) Frequency of SEZ-association and (**D and F**) oxidative burst in SEZ-associated porcine blood granulocytes. Bars and error bars represent mean values and standard deviations, respectively. In E and F, recombinant SzM_C33 was added to whole blood of six piglets prior to incubation with 2 × 10^6^ CFU/mL SEZ. Statistical analysis was performed using RM one-way ANOVA and Šídák’s multiple comparison test. Significant differences are indicated (Not significant [ns] *P* ≥ 0.05, *P < 0.05, **P < 0.01, ***P < 0.001, **** *P* < 0.0001)

We hypothesized that SzM expression protects SEZ strain C33 against phagocytosis, explaining its important role in bacterial survival in the blood. Using flow cytometry, we gated granulocytes using forward scatter (FSC) and side scatter (SSC) and verified this strategy by labeling porcine blood leukocytes with the markers CD14 and SWC8 to distinguish lymphocytes, monocytes, basophils, and eosinophils from neutrophils (54). The results showed that only a few monocytes might be gated, along with granulocytes (Fig. S3). Accordingly, the use of FSC and SSC for granulocyte gating was continued throughout the following assays. Subsequently, we measured the association of Cell Trace FarRed-labeled streptococci with granulocytes and determined the oxidative burst via dihydrorhodamine 123 (DHR123) staining as an indicator of neutrophil activation. As this investigation does not allow us to distinguish between intracellular streptococci or those bound to the outer surface of the granulocytes, we refer to the FarRed-positive granulocytes as “associated with SEZ,” with this term including both bound and internalized SEZ. Figure 3A shows the gating of granulocytes and a negative control sample, whereas Fig. 3B shows exemplary results in the reconstituted blood of one animal. In contrast to our expectation, the association with granulocytes (Fig. 3C) and the associated oxidative burst (Fig. 3D) were significantly enhanced in the SEZ strain C33 WT (19% ± 2.3% SD) in comparison to its isogenic deletion mutant C33Δ*szm* (2.9% ± 0.6% SD). Furthermore, SEZ C33 WT showed a significantly higher association with ROS-generating granulocytes than SEZ C2 (7% ± 0.8% SD). The association of SEZ strain C2 and its isogenic mutant C2Δ*szm* with porcine neutrophils was comparable (WT: 8.9% ± 1% SD, mutant: 7.1% ± 1.4% SD) although the oxidative burst in the associated neutrophils was reduced for the mutant strain C2Δ*szm* (2.4% ± 0.4% SD).

Next, we repeated the oxidative burst experiment with the addition of recombinant SzM_C33 to investigate whether this could reverse the phenotype of SEZ C33 by hindering its association with granulocytes or whether it enhances the association of C33Δ*szm*. Neither for the WT C33 nor for the *szm* mutant, the recombinant protein resulted in a significant change in the number of granulocytes associated with SEZ (Fig. 3E, WT: 11% ± 1.6% SD vs 12.2% ± 1.6% SD and mutant: 0.9% ± 0.1% SD vs 0.7% ± 0.1% SD), but in both cases, it caused a significantly higher activation rate, and more of the granulocytes associated with SEZ performed an oxidative burst (Fig. 3F, WT: 6.1% ± 1.2% SD vs 9.4% ± 1.8% SD and mutant: 0.1% ± 0.05% SD vs 0.2% ± 0.06% SD). In conclusion, SzM_C33 expression on the surface of WT SEZ mediates the association of streptococci with porcine granulocytes, which is not reversed by recombinant SzM_C33 in solution. Both recombinant SzM and SzM produced by WT SEZ C33 triggered an oxidative burst in these cells.

### SzM_C33 mediates phagocytosis and intracellular survival of SEZ in porcine blood

**Fig 4:**
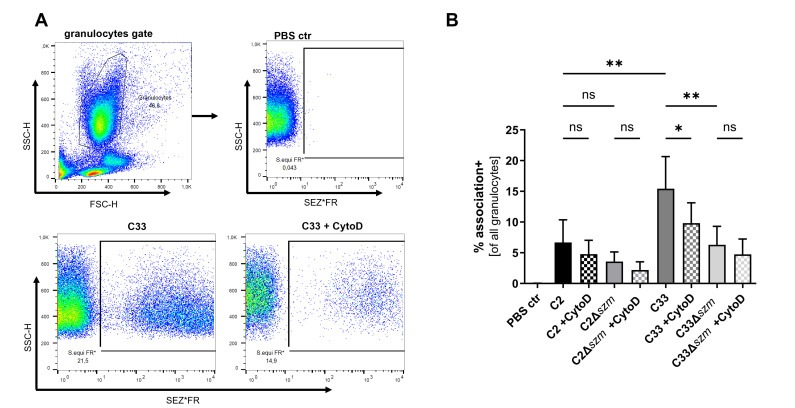
Cytochalasin D (Cyto D) reduces the association of *S. equi* subsp. *zooepidemicus* C33 with porcine granulocytes. (**A**) Flow cytometry gating strategy to measure the frequency of granulocytes associated with SEZ labeled with Cell Trace FarRed. (**B**) Inhibition of association of the SEZ strains C2 and C33 and their isogenic *szm* deletion mutants with porcine blood granulocytes by Cyto D. Sera collected from 6-week-old weaning piglets (*n* = 6) were used to reconstitute porcine blood, which was incubated with Cyto D (10 µg/mL) or PBS and subsequently infected with 2 × 10^6^ CFU/mL of Cell Trace FarRed-labeled streptococci. Bars and error bars represent mean values and standard deviations, respectively. Statistical analysis was performed using RM one-way ANOVA and Šídák’s multiple comparison test. Significant differences are indicated (Not significant (ns) *P* ≥ 0.05, *P < 0.05, **P < 0.01).

The results in Fig. 3 show that SzM_C33 is important for the association of SEZ with granulocytes in SEZ-infected blood. However, our flow cytometric analysis did not allow the distinction of granulocytes with streptococci bound to the outside of the cell from those with SEZ located intracellularly although the increased oxidative burst determined by Rho 123 staining is in accordance with phagocytosis. We inhibited phagocytosis by adding Cytochalasin D (Cyto D) to clarify whether SEZ C33 is phagocytosed by granulocytes in porcine blood. As shown in Fig. 4, Cyto D significantly reduced the association between SEZ C33 and porcine granulocytes. Notably, the association of SEZ C2 and *szm* mutants with granulocytes was also lower in the presence of Cyto D, but the differences were not significant.

**Fig 5:**
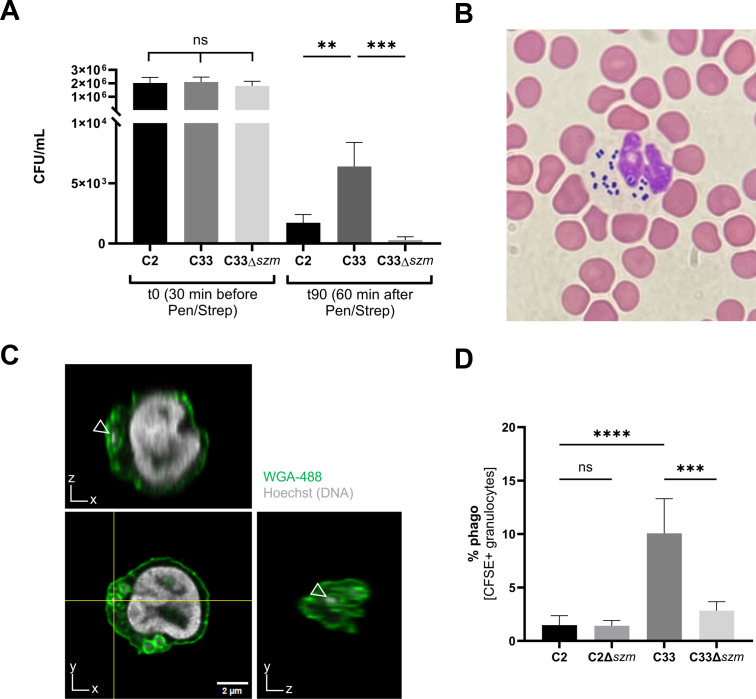
SzM_C33 expression mediates survival of SEZ during penicillin/streptomycin treatment of porcine blood (**A**) in agreement with intracellular detection of WT streptococci in porcine blood neutrophils (**B–D**). A Porcine blood of 6-week-old piglets (*n* = 6) was infected with 2 × 10^6^ CFU/mL of the indicated SEZ WT strain (C33 or C2) or the isogenic *szm* deletion mutant of SEZ C33. Thirty minutes after infection 60 µg/mL penicillin plus 100 µg/mL streptomycin were added as indicated, and blood was incubated at 37°C for further 60 min (T90). After centrifugation and washing of blood cells, their lysis was induced through resuspension in deionized, sterilized water and SEZ were plated. (**B**) Diff Quick staining of a blood sample infected with SEZ C33. The image was captured using an OLYMPUS AX70 microscope. (**C**) Fluorescence analysis of blood infected with SEZ C33 WT after staining with wheat germ agglutinin (WGA)-Alexa-Fluor 488 (WGA-488). The latter was applied at 1:100 dilution to visualize the streptococcal cell walls and the membrane of the eukaryotic cells. The DNA of the bacteria and cells was stained with Hoechst (gray). Orthogonal views of the position indicated by the yellow lines in the *xy* view are shown. Images were captured on a Leica Stellaris 8 FALCON confocal laser scanning microscope. (**D**) Porcine blood (*n* = 9) was infected with 2 × 10^6^ CFU/mL of Cell Trace CFSE-labeled streptococci for 30 min. Frequency of granulocytes showing CFSE signal was measured by flow cytometry (BD LSRFortessa) after the addition of 1.2 mg/mL trypan blue and gating on SSC^high^ granulocytes as shown in Fig. 3 and 4. Statistical analysis was performed using RM one-way ANOVA and Šídák’s multiple comparison test. Relevant comparisons are indicated (Not significant (ns) *P* ≥ 0.05, *P < 0.05, **P < 0.01, ***P < 0.001, **** *P* < 0.0001).

Intracellular survival of streptococci in host cells is associated with protection against antibiotics, such as gentamicin, penicillin, and streptomycin, which poorly penetrate the intracellular milieu (55–58). We measured the survival of SEZ C2, C33, and C33Δ*szm* in porcine blood after the treatment of blood 30 min after infection with penicillin and streptomycin for 60 min. Both strains were highly sensitive to penicillin, as determined by disk diffusion assay. After 60 min of antibiotic treatment, with subsequent washing and lysis of the blood cells, a significantly higher number of C33 WT bacteria were recovered compared to its isogenic *szm* mutant and C2 WT (Fig. 5A C33: 6.4 × 10^3^ ± 2.0 × 10^3^ CFU/mL SD vs C33Δ*szm*: 0.3 × 10^3^ ± 0.3 × 10^3^ CFU/mL SD or C2: 1.7 × 10^3^ ± 0.7 × 10^3^ CFU/mL SD). The bactericidal effect of the combined penicillin and streptomycin treatment was verified by plating serial dilutions of plasma obtained from treated samples. The localization of streptococci in porcine blood granulocytes was confirmed by Diff Quick staining of blood smears at the end of the assay (Fig. 5B). Wheat germ agglutinin (WGA) binds to N-acetyl-D-glucosamine present in the cell wall of bacteria and glycoproteins on the surface of eukaryotic cells. Fluorescence analysis of SEZ C33 infected blood with WGA-Alexa Fluor 488 staining generated signals that were consistent with intracellular streptococci (Fig. 5C).

To further test whether SEZ C33 is localized inside granulocytes, we conducted an experiment that allows quenching of fluorescence emitted by extracellular streptococci. For this, streptococci were labeled with Cell Trace CFSE before the addition to the blood of 7-week-old piglets. Compared to blood incubated with SEZ C2 or the *szm* mutant C33Δ*szm*, a significantly higher number of granulocytes showed the SEZ*CFSE signal when incubated with SEZ C33 after quenching of extracellular signals with trypan blue (Fig. 5D, C33: 10.08% ± 3.2% SD vs C33Δ*szm*: 2.84% ± 0.84% SD or C2: 1.48% ± 0.88% SD).

In conclusion, Cyto D and penicillin/streptomycin protection experiments, microscopy, and quenching analysis indicated SzM_C33-mediated internalization of SEZ in porcine blood granulocytes and intracellular survival in blood infected with SEZ C33 *in vitro*.

### SzM_C33 mediates survival in purified porcine granulocytes

**Fig 6:**
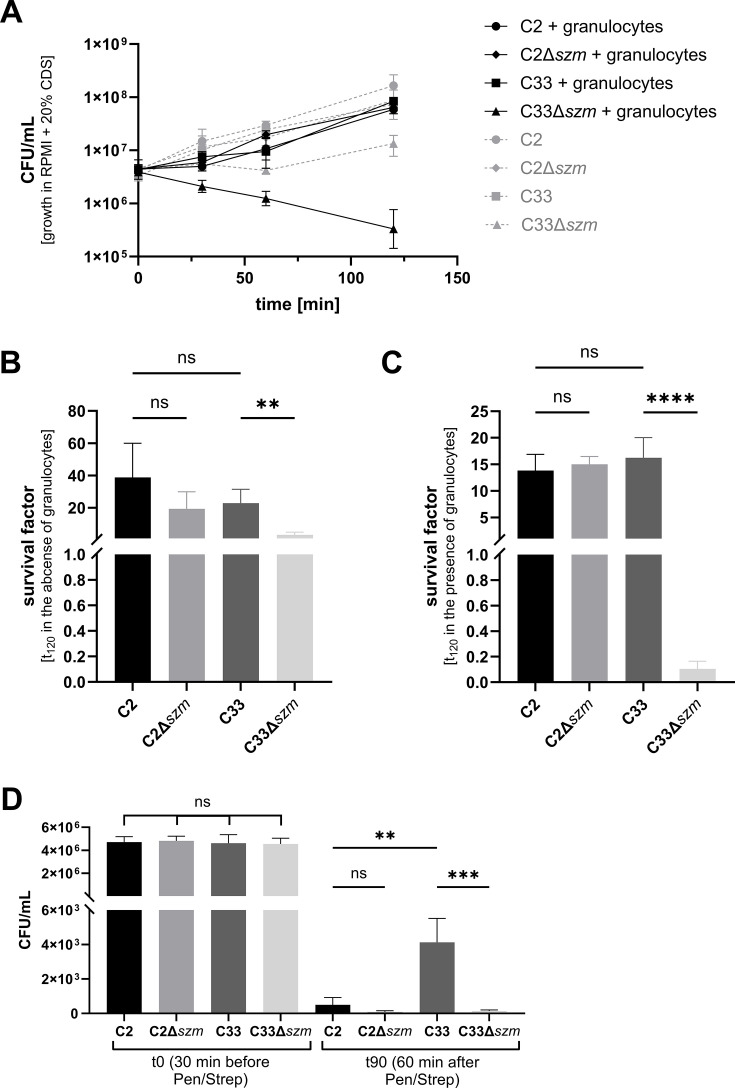
In the presence of serum lacking specific immunoglobulins, SzM_C33 but not SzM_C2 expression is necessary for the proliferation of the respective WT strain, especially in the presence of purified porcine granulocytes. (**A) **Growth and (**B and C**) survival pattern of the indicated SEZ strains in the absence (**A and B**) or presence (**A and C**) of purified porcine granulocytes (MOI 1:2) and sera drawn from colostrum-deprived piglets (CDS) after an incubation period of up to 120 min. (**D**) In an additional experiment, samples with SEZ-infected granulocytes were taken after 30 min of incubation and treated with penicillin (60 µg/mL) and streptomycin (100 µg/mL) for 60 min, followed by washing and lysis of cells and plating of the remaining SEZ. Granulocytes were obtained from pigs aged between 4 and 9 weeks. Statistical analysis was performed using RM one-way ANOVA and Šídák’s multiple comparison test (**B and C**: *n* = 9, D: *n* = 6). Significant differences are indicated (Not significant (ns) *P* ≥ 0.05, *P < 0.05, **P < 0.01, ****P < 0.0001).

The experiments shown in Fig. 2 to 5 were conducted with SEZ-infected blood from piglets. As shown in Fig. 2B and C, this blood includes different immunoglobulins that bind to the SEZ. We also expect nonspecific IgG-Fc binding under these conditions. Serum of colostrum-deprived piglets (CDS) collected immediately after birth lacks specific immunoglobulins as piglets receive maternal antibodies only via colostrum. Purified porcine granulocytes were infected with SEZ C2 and C33 and their *szm* deletion mutants in the presence of RPMI medium with 20% CDS. As shown in Fig. 6A, both WT strains proliferated under these conditions. However, SzM_C2 expression was not necessary for this phenotype of C2 WT, as C2Δ*szm* growth was not attenuated. In contrast, C33Δ*szm* was efficiently killed (Fig. 6A and C). This is in accordance with the functionality of granulocytes under these conditions, as C33Δ*szm* proliferated slightly in RPMI medium with 20% CDS in the absence of granulocytes (Fig. 6A and B). The addition of penicillin and streptomycin 30 min after infection of purified granulocytes with the different SEZ strains revealed significantly higher numbers of viable SEZ C33 bacteria inside the granulocytes in comparison to SEZ C2 and C33Δ*szm* (Fig. 6D). These results indicate that SzM_C33 mediates a distinct phenotype in the interaction with porcine granulocytes, most likely independent of IgG Fc and fibrinogen binding.

### SEZ C33 causes morbidity in piglets after experimental infection

Finally, we wanted to confirm that strain SEZ C33 is virulent in pigs and that intracellular survival occurs *in vivo*. Experimental intravenous infection of six piglets with 1.5 × 10^7^ CFU of SEZ C33 resulted in morbidity in all animals within the following days (Tables S3 and S4). Clinical signs included fever and reduced appetite but no organ-specific signs of disease. At 12 and 24 h post-infection, heparin blood was drawn from all animals to detect bacteremia *in vivo*. Single colonies of SEZ were detected in the blood of two of the six infected piglets (#890 and #716) at 12 h post-infection. At 24 h post-infection, the piglets showed prominent leukocytosis (Fig. S4).

On the 12th day post experimental infection, one infected piglet (#684) showed signs of central nervous system dysfunction and severe lameness. The piglet was euthanized immediately after the confirmation of these clinical signs. The six mock-infected piglets were free of any clinical signs of disease during an observation period of 14 days. Fibrino-suppurative lesions were not detected in any of the piglets at the end of the observation period, except for the one reaching humane endpoints at 12 dpi (see below).

**Fig 7:**
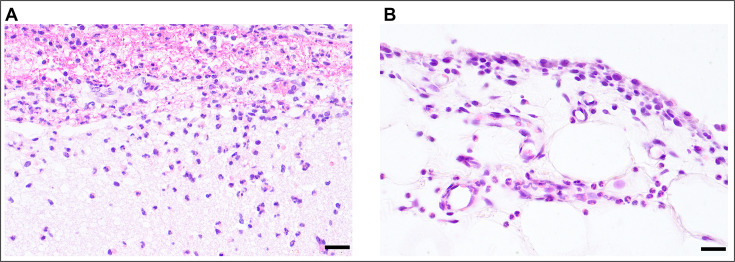
Histopathological findings in a piglet experimentally infected with SEZ C33 and humanely killed 12 dpi due to signs of severe central nervous system dysfunction. SEZ was culturally detected in cerebrospinal fluid (CSF) with 1.7 × 10^4^ CFU/mL. (**A**) Severe acute fibrino-suppurative meningoencephalitis of the cerebral cortex; (**B**) mild acute, suppurative arthritis in one tarsal joint. Hematoxylin–Eosin. Bars A, B = 20 µm.

SEZ C33 was detected in the blood, cerebrospinal fluid, and different internal organs (liver, lung, brain, and spleen) of the piglet, showing central nervous system dysfunction at 12 dpi. Pathohistological screening revealed severe acute diffuse fibrino-suppurative meningoencephalitis and mild acute suppurative arthritis in the tarsal joint (Fig. 7). An aliquot of cerebrospinal fluid (CSF) from this animal was treated *ex vivo* with 10 µg/mL penicillin for 30 min. After centrifugation, threefold washing, and lysis of cells, SEZ was still detectable at 2.8 × 10^3^ CFU/mL (with regard to the original volume of CSF). These results suggest intracellular survival of SEZ C33 in the CSF *in vivo*.

## DISCUSSION

SEZ is an important porcine pathogen, but only a few experimental studies on SEZ in pigs have been published. Finatto et al. recently showed that an SEZ strain originally isolated from a pig with septicemia might efficiently breach the mucosal barrier within a few hours of oral administration (59). Here, we describe a case of meningoencephalitis after intravenous administration of a zoonotic SEZ strain. Together, these studies demonstrate that virulent SEZ strains may cross different host barriers in healthy piglets under experimental conditions, which has only been shown for a limited number of bacterial pathogens. Although pigs harbor various species within the genus *Streptococcus* on their tonsils (60, 61) and mucosal surfaces (62, 63), only *S. suis*, SEZ, and, to a lesser extent, *Streptococcus dysgalactiae* subsp. *equisimilis* (64) have been repeatedly detected as invasive pathogens causing disease outbreaks. SEZ and *S. suis* use very different virulence factors to survive in porcine blood, as *S. suis* does not express an M or M-like protein (65), which is, however, important for the survival of SEZ, as shown in this study (Fig. 2).

This study was initiated by the finding that SEZ C33 proliferates in the blood of 10-week-old conventional piglets. Based on our experience with bactericidal assays using different invasive bacterial pathogens, this is a distinct characteristic. For example, an invasive porcine *Klebsiella pneumoniae* strain proliferates in the blood of suckling and weaned piglets but is killed in the blood of 10-week-old growers (66). Furthermore, different *S. suis* pathotypes survive and proliferate in the blood of younger piglets but are killed in the blood of 8- or 10-week-old piglets (51, 53). This distinct phenotype of SEZ C33 is associated with survival in activated neutrophilic granulocytes. Flow cytometric analysis revealed that SEZ C33 was largely associated with granulocytes in porcine blood. Intracellular survival and localization in granulocytes were demonstrated in this study using the penicillin/streptomycin protection assay, microscopic visualization, and quenching of extracellular SEZ signals by flow cytometry. Notably, the number of viable intracellular streptococci was relatively low after penicillin/streptomycin treatment (Fig. 5A and 6D), even those of SEZ C33, despite the high number of granulocytes showing the SEZ*CFSE signal in the quenching assay. This is most likely due to a certain unavoidable penetration of streptomycin and a bactericidal effect on intracellular bacteria, as shown in investigations of intracellular *Staphylococcus aureus* (67). For this reason, we added only penicillin in the first set of experiments, including the investigation of CSF from the piglet with meningoencephalitis. However, further experiments revealed that penicillin alone did not exhibit sufficient bactericidal activity on extracellular bacteria in a feasible time span, and a high dose of penicillin plus streptomycin was needed to ensure no remaining extracellular survival. This shows the limitations of antibiotic protection assays, as it is difficult to neither accidentally count extracellular bound bacteria nor diminish intracellular bacterial viability. Accordingly, further animal experiments are needed to address the intracellular survival of SEZ C33 *in vivo*.

The second SEZ strain investigated in this study was isolated from a horse with recurrent airway obstruction. To improve the interpretation of our results, we compared the human isolate C33 with the equine isolate C2 after whole-genome sequencing and TEM analysis of capsule thickness. Genome analysis revealed an ANI of 97.22%, which exceeds the proposed species threshold of 96% (68) but is relatively low for isolates assigned to the same subspecies. By comparison, ANI values of ≥98% have been suggested for subspecies discrimination in *Salmonella* (69). This result highlights the considerable genomic diversity within SEZ and matches with ANI values found by Kanyó and Molnár (70). More than 700 genes were identified as strain-specific, being present in only one of the two isolates, which also belonged to distinct sequence types. This extensive genomic divergence suggests that the phenotypic differences observed between the strains cannot be readily attributed to a single protein variant. Consequently, to specifically assess the contribution of SzM, each wild-type strain was investigated in comparison with its respective isogenic Δ*szm* mutant. Though our results demonstrate that the expression of SzM_C33 in SEZ C33 is necessary for survival in porcine blood and inside granulocytes, they do not exclude that further proteins of SEZ C33 are important for intracellular survival.

As described in our previous study, the mature SzM proteins of SEZ exhibit a high degree of sequence diversity. This variability is particularly pronounced within the N-terminal region. Phylogenetic analysis based on the first 216 amino acids demonstrated that SzM_C33 clusters with SzM variants derived from human isolates, whereas SzM_C2 does not group within any distinct phylogenetic cluster (24). Accordingly, our studies on SEZ demonstrate two major differences in SzM-mediated host pathogen interactions between SEZ C2 and C33. First, only SEZ strain C33 binds large amounts of human fibrinogen through SzM expression (24). Second, SEZ strain C2 recruits C1q to the bacterial surface independent of immunoglobulins and without subsequent activation of the complement cascade. The latter has been shown for human and equine C1q (25). Our previous data suggested that both mechanisms are involved in complement evasion. In this study, we demonstrate SzM_C33-mediated survival of SEZ C33 in porcine blood and in purified blood granulocytes in association with uptake by and activation of porcine neutrophils. The SzM-mediated association with neutrophils is most likely independent of fibrinogen binding, as SEZ C33 recruits lower amounts of porcine fibrinogen to the bacterial surface in comparison to SEZ strains C2 and C27 (Fig. S5), and we found higher numbers of SEZ C33 than C2 or the *szm* deletion mutants inside purified porcine granulocytes in the presence of RPMI medium and 20% serum (Fig. 5D). Interestingly, SEZ strain C2 is capable of producing a markedly thick hyaluronic acid capsule—evident from the mucoid colony morphology on blood agar plates and confirmed by STEM analysis of cultures grown in THY broth. Nevertheless, it is the human isolate C33 that shows higher survival factors in porcine blood *in vitro* and SzM-dependent survival in neutrophils. These findings underline the critical contribution of the SzM protein to immune evasion and virulence, independent of the extent of capsular expression. Due to the substantial sequence divergence between the two SzM proteins investigated in this study, as illustrated by the Clustal Omega alignment in Fig. S1, it is currently not possible to identify the specific protein region responsible for the ability of SEZ C33, but not SEZ C2, to survive within activated neutrophils. Further studies employing targeted mutagenesis or domain-swapping approaches will be required to determine which structural elements of SzM contribute to this phenotype.

Medina et al. (71) demonstrated that *S. pyogenes* can survive and disseminate within murine polymorphonuclear cells (PMNs) by transferring infected gentamicin-treated PMNs to naïve mice. In infected PMNs, *S. pyogenes* M23 may breach the phagocytic vacuole and escape into the cytoplasm (57). Another group showed that *S. pyogenes* M1 targets neutrophilic granulocytes in the human blood and survives in human neutrophils through protein M1-mediated inhibition of the fusion of azurophilic granules with phagosomes (72, 73). Nevertheless, *S. pyogenes* M1 infected human neutrophils also show the induction of an oxidative burst (72). Accordingly, GAS M1 and SEZ C33 may use a similar mechanism to survive in blood neutrophils.

To date, there have been no reports describing the survival of SEZ in phagocytes. Equine SEZ strains were shown to survive *in vitro* in HEp-2 and HeLa cell lines(55). Phagosome-like compartments contain SEZ and fuse with lysosomes to form phagolysosomes(55). This study demonstrates the survival of SEZ C33 and its proliferation in the blood in association with activated porcine granulocytes. As this phenotype was not observed for SEZ C2, we postulate that the survival in granulocytes is a phenotype associated with a specific pathotype of SEZ. Experimental infection of piglets with SEZ C33 confirmed the virulence of this strain in an important natural host of SEZ. Furthermore, the detection of culturable SEZ in the CSF of one piglet after treatment with penicillin suggests intracellular survival although further experiments are crucial to confirm that SEZ C33 enters the CSF compartment and causes infection of the central nervous system through neutrophil infection. We did not investigate the infection of piglets with the isogenic SEZ C33 *szm* deletion mutant, but we expect a strong attenuation of this mutant in an infection model, given the fact that the deletion mutant is rapidly killed in porcine blood (Fig. 2).

Infection of human neutrophils with *S. pyogenes* induces the generation of H_2_O_2_ by NADPH oxidase, as determined by measuring the increase in green fluorescence caused by the oxidation of DHR 123 to rhodamine 123 by flow cytometry (72). Cytochalasin B inhibits this fluorescence, suggesting that phagocytosis is important for the induction of oxidative burst (72). Our data (Fig. 3) also showed a strong induction of oxidative burst in granulocytes after infection of porcine blood with SEZ C33. Importantly, this induction depended on SzM_C33 expression and was partially reduced by cytochalasin D (Fig. 4). These results are in agreement with the fact that granulocytes activate NADPH oxidase upon phagocytosis of SEZ C33. However, it is unclear whether SEZ C33 is exposed to an oxidative burst in the phagolysosomes of blood granulocytes. Further studies are required to elucidate the intracellular trafficking and SzM-mediated survival mechanism of SEZ C33 in porcine neutrophilic granulocytes.

## Data Availability

Research data supporting this publication are available from a Zenodo repository (https://doi.org/10.5281/zenodo.20746704).

## SUPPLEMENTAL MATERIAL

Supplemental material iai.00113-26-s0001.pdf Fig. S1 to S5; Tables S1 to S4.

ASM does not own the copyrights to Supplemental Material that may be linked to, or accessed through, an article. The authors have granted ASM a non-exclusive, world-wide license to publish the Supplemental Material files. Please contact the corresponding author directly for reuse.

## References

[1] Reuss SM, Giguère S. 2015. Update on bacterial pneumonia and pleuropneumonia in the adult horse. Vet Clin North Am Equine Pract 31:105–120. doi:10.1016/j.cveq.2014.11.002 25600453

[2] Waller AS. 2014. Equine respiratory disease: a causal role for *Streptococcus zooepidemicus*. Vet J 201:3–4. doi:10.1016/j.tvjl.2014.04.013 24837551

[3] Lindahl SB, Aspán A, Båverud V, Paillot R, Pringle J, Rash NL, Söderlund R, Waller AS. 2013. Outbreak of upper respiratory disease in horses caused by *Streptococcus equi* subsp. *zooepidemicus* ST-24. Vet Microbiol 166:281–285. doi:10.1016/j.vetmic.2013.05.006 23773239

[4] Björnsdóttir S, Harris SR, Svansson V, Gunnarsson E, Sigurðardóttir ÓG, Gammeljord K, Steward KF, Newton JR, Robinson C, Charbonneau ARL, et al.. 2017. Genomic dissection of an icelandic epidemic of respiratory disease in horses and associated zoonotic cases. mBio 8:e00826-17. doi:10.1128/mBio.00826-17 28765219 PMC5539424

[5] Pesavento PA, Hurley KF, Bannasch MJ, Artiushin S, Timoney JF. 2008. A clonal outbreak of acute fatal hemorrhagic pneumonia in intensively housed (shelter) dogs caused by *Streptococcus equi* subsp. *zooepidemicus*. Vet Pathol 45:51–53. doi:10.1354/vp.45-1-51 18192575

[6] FitzGerald W, Crowe B, Brennan P, Cassidy JP, Leahy M, McElroy MC, Casey M, Waller A, Mitchell C. 2017. Acute fatal haemorrhagic pneumonia caused by *Streptococcus equi zooepidemicus* in greyhounds in Ireland with subsequent typing of the isolates. Vet Rec 181:119. doi:10.1136/vr.104275 28600445

[7] Priestnall S, Erles K. 2011. *Streptococcus zooepidemicus*: an emerging canine pathogen. Vet J 188:142–148. doi:10.1016/j.tvjl.2010.04.028 20570190 PMC7110628

[8] Pisoni G, Zadoks RN, Vimercati C, Locatelli C, Zanoni MG, Moroni P. 2009. Epidemiological investigation of *Streptococcus equi* subspecies *zooepidemicus* involved in clinical mastitis in dairy goats. J Dairy Sci 92:943–951. doi:10.3168/jds.2008-1548 19233787

[9] Corpa JM, Carvallo F, Anderson ML, Nyaoke AC, Moore JD, Uzal FA. 2018. *Streptococcus equi* subspecies *zooepidemicus* septicemia in alpacas: three cases and review of the literature. J Vet Diagn Invest 30:598–602. doi:10.1177/1040638718772071 29717639 PMC6505895

[10] Sharp MW, Prince MJ, Gibbens J. 1995. *S zooepidemicus* infection and bovine mastitis. Vet Rec 137:128. doi:10.1136/vr.137.5.128-b 8533263

[11] Las Heras A, Vela AI, Fernández E, Legaz E, Domínguez L, Fernández-Garayzábal JF. 2002. Unusual outbreak of clinical mastitis in dairy sheep caused by *Streptococcus equi* subsp. *zooepidemicus*. J Clin Microbiol 40:1106–1108. doi:10.1128/JCM.40.3.1106-1108.2002 11880454 PMC120234

[12] Hau SJ, Lantz K, Stuart KL, Sitthicharoenchai P, Macedo N, Derscheid RJ, Burrough ER, Robbe-Austerman S, Brockmeier SL. 2022. Replication of *Streptococcus equi* subspecies *zooepidemicus* infection in swine. Vet Microbiol 264:109271. doi:10.1016/j.vetmic.2021.109271 34826647

[13] Chen X, Resende-De-Macedo N, Sitthicharoenchai P, Sahin O, Burrough E, Clavijo M, Derscheid R, Schwartz K, Lantz K, Robbe-Austerman S, et al.. 2020. Genetic characterization of *Streptococcus equi* subspecies *zooepidemicus* associated with high swine mortality in the United States. Transbound Emerg Dis 67:2797–2808. doi:10.1111/tbed.13645 32460392

[14] Costa M de O, Lage B. 2020. *Streptococcus equi* subspecies *zooepidemicus* and sudden deaths in Swine, Canada. Emerg Infect Dis 26:2522–2524. doi:10.3201/eid2610.191485 PMC7510724 32946731

[15] Garmyn A, Van de Velde N, Braeckmans D, Ronsmans S, Boyen F, Verlinden M. 2020. An outbreak associated with *Streptococcus equi* subsp. *zooepidemicus* in layers: evidence of fecal transmission. Avian Dis 64:343–346. doi:10.1637/aviandiseases-D-19-00191 33205184

[16] Soedarmanto I, Pasaribu FH, Wibawan IWT, Lämmler C. 1996. Identification and molecular characterization of serological group C streptococci isolated from diseased pigs and monkeys in Indonesia. J Clin Microbiol 34:2201–2204. doi:10.1128/jcm.34.9.2201-2204.1996 8862585 PMC229217

[17] Pelkonen S, Lindahl SB, Suomala P, Karhukorpi J, Vuorinen S, Koivula I, Väisänen T, Pentikäinen J, Autio T, Tuuminen T. 2013. Transmission of *Streptococcus equi* subspecies *zooepidemicus* infection from horses to humans. Emerg Infect Dis 19:1041–1048. doi:10.3201/eid1907.121365 23777752 PMC3713971

[18] Edwards AT, Roulson M, Ironside MJ. 1988. A milk-borne outbreak of serious infection due to *Streptococcus zooepidemicus* (Lancefield group C). Epidemiol Infect 101:43–51. doi:10.1017/s0950268800029204 3402548 PMC2249326

[19] Kim M, Heo ST, Oh H, Kim M, Jo J, Kim YR, Lee KH, Yoo JR. 2022. Human zoonotic infectious disease caused by *Streptococcus equi* subsp. *zooepidemicus*. Zoonoses Public Health 69:136–142. doi:10.1111/zph.12895 34585529

[20] Feng H. 1977. Outbreak of swine *Streptococcosis* in Sichan province and identification of pathogen. Anim Husbandry Vet Med Lett 2:7–12.

[21] Ma Z, Geng J, Yi L, Xu B, Jia R, Li Y, Meng Q, Fan H, Hu S. 2013. Insight into the specific virulence related genes and toxin-antitoxin virulent pathogenicity islands in swine *Streptococcosis* pathogen *Streptococcus equi* ssp. *zooepidemicus* strain ATCC35246. BMC Genomics 14:377. doi:10.1186/1471-2164-14-377 23742619 PMC3750634

[22] Sitthicharoenchai P, Derscheid R, Schwartz K, Macedo N, Sahin O, Chen X, Li G, Main R, Burrough E. 2020. Cases of high mortality in cull sows and feeder pigs associated with *Streptococcus equi* subsp. *zooepidemicus* septicemia. J Vet Diagn Invest 32:565–571. doi:10.1177/1040638720927669 32532177 PMC7438661

[23] Timoney JF, Walker J, Zhou M, Ding J. 1995. Cloning and sequence analysis of a protective M-like protein gene from *Streptococcus equi* subsp. *zooepidemicus*. Infect Immun 63:1440–1445. doi:10.1128/iai.63.4.1440-1445.1995 7890407 PMC173172

[24] Bergmann René, Jentsch M-C, Uhlig A, Müller U, van der Linden M, Rasmussen M, Waller A, von Köckritz-Blickwede M, Baums CG. 2019. Prominent binding of human and equine fibrinogen to *Streptococcus equi* subsp. *zooepidemicus* is mediated by specific SzM types and is a distinct phenotype of zoonotic isolates. Infect Immun 88:e00559-19. doi:10.1128/IAI.00559-19 31636136 PMC6921669

[25] Bergmann R, Schroedl W, Müller U, Baums CG. 2023. A distinct variant of the SzM protein of *Streptococcus equi* subsp. *zooepidemicus* recruits C1q independent of IgG binding and inhibits activation of the classical complement pathway. Virulence 14:2235461. doi:10.1080/21505594.2023.2235461 37450582 PMC10351459

[26] Hammerschmidt S, Wolff S, Hocke A, Rosseau S, Müller E, Rohde M. 2005. Illustration of pneumococcal polysaccharide capsule during adherence and invasion of epithelial cells. Infect Immun 73:4653–4667. doi:10.1128/IAI.73.8.4653-4667.2005 16040978 PMC1201225

[27] Abueg LAL, Afgan E, Allart O, Awan AH, Bacon WA, Baker D, Bassetti M, Batut B, Bernt M, Blankenberg D, et al.. 2024. The Galaxy platform for accessible, reproducible, and collaborative data analyses: 2024 update. Nucleic Acids Res 52:W83–W94. doi:10.1093/nar/gkae410 38769056 PMC11223835

[28] de Sena Brandine G, Smith AD. 2021. Falco: high-speed FastQC emulation for quality control of sequencing data. F1000Res 8:1874. doi:10.12688/f1000research.21142.2 PMC7845152 33552473

[29] Bolger AM, Lohse M, Usadel B. 2014. Trimmomatic: a flexible trimmer for illumina sequence data. Bioinformatics 30:2114–2120. doi:10.1093/bioinformatics/btu170 24695404 PMC4103590

[30] Wick RR, Judd LM, Gorrie CL, Holt KE. 2016. Unicycler: resolving bacterial genome assemblies from short and long sequencing reads. bioRxiv. doi:10.1101/096412 PMC5481147 28594827

[31] Jain C, Rodriguez-R LM, Phillippy AM, Konstantinidis KT, Aluru S. 2018. High throughput ANI analysis of 90K prokaryotic genomes reveals clear species boundaries. Nat Commun 9:5114. doi:10.1038/s41467-018-07641-9 30504855 PMC6269478

[32] Seemann T. 2014. Prokka: rapid prokaryotic genome annotation. Bioinformatics 30:2068–2069. doi:10.1093/bioinformatics/btu153 24642063

[33] Cuccuru G, Orsini M, Pinna A, Sbardellati A, Soranzo N, Travaglione A, Uva P, Zanetti G, Fotia G. 2014. Orione, a web-based framework for NGS analysis in microbiology. Bioinformatics 30:1928–1929. doi:10.1093/bioinformatics/btu135 24618473 PMC4071203

[34] Page AJ, Cummins CA, Hunt M, Wong VK, Reuter S, Holden MTG, Fookes M, Falush D, Keane JA, Parkhill J. 2015. Roary: rapid large-scale prokaryote pan genome analysis. Bioinformatics 31:3691–3693. doi:10.1093/bioinformatics/btv421 26198102 PMC4817141

[35] Torsten S. 2016. MLST: scan contig files against PubMLST typing schemes. GitHub. https://github.com/tseemann/mlst.

[36] Danecek P, Bonfield JK, Liddle J, Marshall J, Ohan V, Pollard MO, Whitwham A, Keane T, McCarthy SA, Davies RM, et al.. 2021. Twelve years of SAMtools and BCFtools. Gigascience 10:giab008. doi:10.1093/gigascience/giab008 33590861 PMC7931819

[37] Li H. 2011. Improving SNP discovery by base alignment quality. Bioinformatics 27:1157–1158. doi:10.1093/bioinformatics/btr076 21320865 PMC3072548

[38] Li H, Durbin R. 2010. Fast and accurate long-read alignment with burrows-wheeler transform. Bioinformatics 26:589–595. doi:10.1093/bioinformatics/btp698 20080505 PMC2828108

[39] Li H, Durbin R. 2009. Fast and accurate short read alignment with burrows–wheeler transform. Bioinformatics 25:1754–1760. doi:10.1093/bioinformatics/btp324 19451168 PMC2705234

[40] Li H. 2013. Aligning sequence reads, clone sequences and assembly contigs with BWA-MEM

[41] Sievers F, Wilm A, Dineen D, Gibson TJ, Karplus K, Li W, Lopez R, McWilliam H, Remmert M, Söding J, Thompson JD, Higgins DG. 2011. Fast, scalable generation of high-quality protein multiple sequence alignments using clustal omega. Mol Syst Biol 7:539. doi:10.1038/msb.2011.75 21988835 PMC3261699

[42] Madeira F, Madhusoodanan N, Lee J, Eusebi A, Niewielska A, Tivey ARN, Lopez R, Butcher S. 2024. The EMBL-EBI job dispatcher sequence analysis tools framework in 2024. Nucleic Acids Res 52:W521–W525. doi:10.1093/nar/gkae241 38597606 PMC11223882

[43] Seele J, Hillermann L-M, Beineke A, Seitz M, von Pawel-Rammingen U, Valentin-Weigand P, Baums CG. 2015. The immunoglobulin M-degrading enzyme of *Streptococcus suis*, IdeSsuis, is a highly protective antigen against serotype 2. Vaccine 33:2207–2212. doi:10.1016/j.vaccine.2015.03.047 25825330

[44] Weiße C, Dittmar D, Jakóbczak B, Florian V, Schütze N, Alber G, Klose K, Michalik S, Valentin-Weigand P, Völker U, et al.. 2021. Immunogenicity and protective efficacy of a *Streptococcus suis* vaccine composed of six conserved immunogens. Vet Res 52:112. doi:10.1186/s13567-021-00981-3 34433500 PMC8390293

[45] Rungelrath V, Öhlmann S, Alber G, Schrödl W, von Köckritz-Blickwede M, de Buhr N, Martens A, Baums CG, Schütze N. 2020. Survival of *Streptococcus* suis in porcine blood is limited by the antibody- and complement-dependent oxidative burst response of granulocytes. Infect Immun 88. doi:10.1128/IAI.00598-19 PMC7035923 31843967

[46] Schindelin J, Arganda-Carreras I, Frise E, Kaynig V, Longair M, Pietzsch T, Preibisch S, Rueden C, Saalfeld S, Schmid B, et al.. 2012. Fiji: an open-source platform for biological-image analysis. Nat Methods 9:676–682. doi:10.1038/nmeth.2019 22743772 PMC3855844

[47] Nuutila J, Lilius EM. 2005. Flow cytometric quantitative determination of ingestion by phagocytes needs the distinguishing of overlapping populations of binding and ingesting cells. Cytometry A 65:93–102. doi:10.1002/cyto.a.20139 15825183

[48] Schütze N, Raue R, Büttner M, Alber G. 2009. Inactivated parapoxvirus ovis activates canine blood phagocytes and T lymphocytes. Vet Microbiol 137:260–267. doi:10.1016/j.vetmic.2009.01.035 19251383

[49] Benga L, Fulde M, Neis C, Goethe R, Valentin-Weigand P. 2008. Polysaccharide capsule and suilysin contribute to extracellular survival of *Streptococcus suis* co-cultivated with primary porcine phagocytes. Vet Microbiol 132:211–219. doi:10.1016/j.vetmic.2008.05.005 18565698

[50] Baums CG, Kaim U, Fulde M, Ramachandran G, Goethe R, Valentin-Weigand P. 2006. Identification of a novel virulence determinant with serum opacification activity in Streptococcus suis. Infect Immun 74:6154–6162. doi:10.1128/IAI.00359-06 17057090 PMC1695488

[51] Rieckmann K, Seydel A, Szewczyk K, Klimke K, Rungelrath V, Baums CG. 2018. *Streptococcus suis* cps7: an emerging virulent sequence type (ST29) shows a distinct, IgM-determined pattern of bacterial survival in blood of piglets during the early adaptive immune response after weaning. Vet Res 49:48. doi:10.1186/s13567-018-0544-8 29903042 PMC6003162

[52] Seidel AM, Kauffold J, Protschka M, Baums CG, Alber G, Eschke M. 2024. Porcine non-conventional B-1-like cells are a potent source of *Streptococcus suis*-binding IgM. Front Immunol 15:1495359. doi:10.3389/fimmu.2024.1495359 39624084 PMC11609192

[53] Mayer L, Bornemann N, Lehnert S, de Greeff A, Strutzberg-Minder K, Rieckmann K, Baums CG. 2021. Survival patterns of *Streptococcus suis* serotypes 1 and 14 in porcine blood indicate cross-reactive bactericidal antibodies in naturally infected pigs. Vet Microbiol 260:109183. doi:10.1016/j.vetmic.2021.109183 34304027

[54] Summerfield A, Haverson K, Thacker E, McCullough KC. 2001. Differentiation of porcine myeloid bone marrow haematopoietic cell populations. Vet Immunol Immunopathol 80:121–129. doi:10.1016/s0165-2427(01)00288-4 11445223

[55] Skive B, Rohde M, Molinari G, Braunstein TH, Bojesen AM. 2017. *Streptococcus equi* subsp. *zooepidemicus* invades and survives in epithelial cells. Front Cell Infect Microbiol 7. doi:10.3389/fcimb.2017.00465 PMC5681531 29164073

[56] Sharma O, O’Seaghdha M, Velarde JJ, Wessels MR. 2016. NAD+-glycohydrolase promotes intracellular survival of group A *Streptococcus*. PLoS Pathog 12:e1005468. doi:10.1371/journal.ppat.1005468 26938870 PMC4777570

[57] Medina E, Rohde M, Chhatwal GS. 2003. Intracellular survival of *Streptococcus* pyogenes in polymorphonuclear cells results in increased bacterial virulence. Infect Immun 71:5376–5380. doi:10.1128/IAI.71.9.5376-5380.2003 12933887 PMC187312

[58] Bongers S, Hellebrekers P, Leenen LPH, Koenderman L, Hietbrink F. 2019. Intracellular penetration and effects of antibiotics on *Staphylococcus aureus* inside human neutrophils: a comprehensive review. Antibiotics (Basel) 8:54. doi:10.3390/antibiotics8020054 31060222 PMC6628357

[59] Finatto AN, Koirala S, Facioli FL, Barbosa JA, Nosach R, Costa M de O. 2024. Organ distribution and early pathogenesis of *Streptococcus equi* subsp. *zooepidemicus* in swine. Comp Immunol Microbiol Infect Dis 104:102095. doi:10.1016/j.cimid.2023.102095 37988858

[60] Baele M, Chiers K, Devriese LA, Smith HE, Wisselink HJ, Vaneechoutte M, Haesebrouck F. 2001. The gram-positive tonsillar and nasal flora of piglets before and after weaning. J Appl Microbiol 91:997–1003. doi:10.1046/j.1365-2672.2001.01463.x 11851806

[61] de Oliveira IMF, Fredriksen S, Gutiérrez MF, Harmsen HJM, Boekhorst J, van Baarlen P, Wells JM. 2025. Culturomics of the pig tonsil microbiome identifies new species and an untapped source of novel antimicrobials. Microbiome 13:86. doi:10.1186/s40168-025-02064-3 40158143 PMC11954270

[62] Wylensek D, Hitch TCA, Riedel T, Afrizal A, Kumar N, Wortmann E, Liu T, Devendran S, Lesker TR, Hernández SB, et al.. 2020. A collection of bacterial isolates from the pig intestine reveals functional and taxonomic diversity. Nat Commun 11:6389. doi:10.1038/s41467-020-19929-w 33319778 PMC7738495

[63] Poor AP, Moreno LZ, Monteiro MS, Matajira CEC, Dutra MC, Leal DF, Silva APS, Gomes VTM, Barbosa MRF, Sato MIZ, et al.. 2022. Vaginal microbiota signatures in healthy and purulent vulvar discharge sows. Sci Rep 12:9106. doi:10.1038/s41598-022-13090-8 35650232 PMC9160009

[64] Oh SI, Kim JW, Kim J, So B, Kim B, Kim HY. 2020. Molecular subtyping and antimicrobial susceptibility of *Streptococcus dysgalactiae* subspecies *equisimilis* isolates from clinically diseased pigs. J Vet Sci 21:e57. doi:10.4142/jvs.2020.21.e57 32735095 PMC7402932

[65] Fittipaldi N, Segura M, Grenier D, Gottschalk M. 2012. Virulence factors involved in the pathogenesis of the infection caused by the swine pathogen and zoonotic agent *Streptococcus suis*. Future Microbiol 7:259–279. doi:10.2217/fmb.11.149 22324994

[66] Krieger AK, Öhlmann S, Mayer L, Weiße C, Rieckmann K, Baums CG. 2022. Porcine iucA+ but rmpA- *Klebsiella pneumoniae* strains proliferate in blood of young piglets but are killed by IgM and complement dependent opsonophagocytosis when these piglets get older. Vet Microbiol 266:109361. doi:10.1016/j.vetmic.2022.109361 35131553

[67] Easmon CSF. 1979. The effect of antibiotics on the intracellular survival of *Staphylococcus aureus in vitro*. Br J Exp Pathol 60:24. 465304 PMC2041428

[68] Ciufo S, Kannan S, Sharma S, Badretdin A, Clark K, Turner S, Brover S, Schoch CL, Kimchi A, DiCuccio M. 2018. Using average nucleotide identity to improve taxonomic assignments in prokaryotic genomes at the NCBI. Int J Syst Evol Microbiol 68:2386–2392. doi:10.1099/ijsem.0.002809 29792589 PMC6978984

[69] Pearce ME, Langridge GC, Lauer AC, Grant K, Maiden MCJ, Chattaway MA. 2021. An evaluation of the species and subspecies of the genus salmonella with whole genome sequence data: proposal of type strains and epithets for novel *S. enterica* subspecies VII, VIII, IX, X and XI. Genomics 113:3152–3162. doi:10.1016/j.ygeno.2021.07.003 34242711 PMC8426187

[70] Kanyó I, Molnár LV. 2016. Procaryotic species and subspecies delineation using average nucleotide identity and gene order conservation. Gene Rep 5:75–82. doi:10.1016/j.genrep.2016.09.004

[71] Medina E, Goldmann O, Toppel AW, Chhatwal GS. 2003. Survival of *Streptococcus* pyogenes within host phagocytic cells: a pathogenic mechanism for persistence and systemic invasion. J Infect Dis 187:597–603. doi:10.1086/373998 12599076

[72] Staali Leïla, Bauer S, Mörgelin M, Björck L, Tapper H. 2006. *Streptococcus* pyogenes bacteria modulate membrane traffic in human neutrophils and selectively inhibit azurophilic granule fusion with phagosomes. Cell Microbiol 8:690–703. doi:10.1111/j.1462-5822.2005.00662.x 16548894

[73] Staali L, Mörgelin M, Björck L, Tapper H. 2003. *Streptococcus* pyogenes expressing M and M-like surface proteins are phagocytosed but survive inside human neutrophils. Cell Microbiol 5:253–265. doi:10.1046/j.1462-5822.2003.00272.x 12675683

