# Supplementary material for: A zoonotic Streptococcus equi subsp. zooepidemicus strain survives in activated blood neutrophils of pigs through SzM expression
Source: Infect Immun. 2026 Jul 15;94(8):e00113-26. doi: 10.1128/iai.00113-26 (PMC13459979; doi:10.1128/iai.00113-26)
Supplement: Supplementary file 1 [file iai.00113-26-s0001.pdf]

# Supplementary Material

## A zoonotic Streptococcus equi subsp. zooepidemicus strain survives in activated blood neutrophils of pigs through SzM expression

Sophie Öhlmann, Christine Weiße, Andreas Nerlich, Tanja Wurzer, Yannick Max Fornoville, Anna Majcher, Thomas Grochow, Simone A. Fietz, Reiner Ulrich, René Bergmann, Christoph Georg Baums

```
CLUSTAL O(1.2.4) multiple sequence alignment


SzM_C2       YSLRRLKKGTASVAVALAVLGAGTASIMTASAQDYDIVDLTDEISRSEHIVNEYHKLLDE      60
SzM_C33      --------ASASVLAAASVLGGTE------------------------------------      16
                     .:*** .* :***.

SzM_C2       ALQLSEHITELQKQEFSVRRSQDPALTEHLKKWNDWATETLGRINGWNTQKALASKVQEL      120
SzM_C33      --------------------------AKAQDHWY-WVEPEYV---------------TEA      34
                                       ::  .:*  *.                     *

SzM_C2       LSEKDKVETLAKEKEGLEKELASQQQQQKQLEQKLQQTDSNMLDLAKFIEEQKNNINSLN      180
SzM_C33      LYARDQ---LIRENAELQ-----------------DLIKSEVIDYNKLVDKKKAV----E      70
             *  :*:   * :*:  *:                 :  .*:::*  *:::::*      :

SzM_C2       KEISSLKDQAELDKETIDMLTDTIEGQDEEISTYIKTL--------KDKEKLLERETEQL      232
SzM_C33      KEL--------------LLTKDSLEATAKDKQALIKAKTRLEDSLLATKDALTKSKEEAQ      116
             **:               : .*::*.  :: .: **:           *: * : : *

SzM_C2       GDLELAYEQQQRVLEDTEQKLTDSQSTIDSLSQKLLTKQDELVNLNQQLTDNKRMYEAFL      292
SzM_C33      GHLEALNHK--------NAQIADLVNKNDDMAAQLSASQER----NAELERNLASYDRLI      164
             *.**   .:        : :::*  .. *.:: :* :.*:.    * :*  *   *: ::

SzM_C2       HQAKEALAKQNQEIDSLKDLDRINRNLLGNAKDELGKLSAKNEQLSQDNAKLEEDQ----      348
SzM_C33      ESAKREMAEKLAEIKRLSAENAD-------SK-------AQIESLQADVAKLTENLASYG      210
             ..**. :*::  **. *.  :         :*       *: *.*. * *** *:

SzM_C2       KVAEANRRGLRRDL---EASREAKKKVEAELADLNTKLDKLEEDQKISEANRKGLHRDLE      405
SzM_C33      RLIESAKREMAEKLAEIETLTKQKAEVEKILAEEQAKVSELEK-----------------      253
             :: *: :* : ..*   *:  : * :**  **: ::*:.:**:

SzM_C2       AAREAKKKVEAELADVNAKLGALEKLNKELEDGKKLSDQEKAELQAKLEAETKALKEQIA      465
SzM_C33      -----------QVEVANAKVTELEKQKAEAEDK--IATLEK-D-LEAIKVENAKYKEQLA      298
                        ::  .***:  *** : * **   ::  ** :    ::.*.   ***:*

SzM_C2       KQAEEIAKLKEEQAKKQKEETPKTPEKPETKPEADPKADKPAKPEAKPAAPKADTKKAAS      525
SzM_C33      KQAEELEKAKAEAEALNKQI--AQLKEELAKKSEAPKADKPAKPEAKPAAPKADTKKAAP      356
             *****: * * *    :*:      ::  :* .  ************************

SzM_C2       KAGQLPSTGESANPFFTIAALTVIAGA      552
SzM_C33      KAGQLPSTGESANP-------------      370
             **************
```

**Suppl. Fig. S1 Alignment of SzM_C2 (AZW7490.1) and SzM_C33 (AZW07476.1)** using clustal omega web service

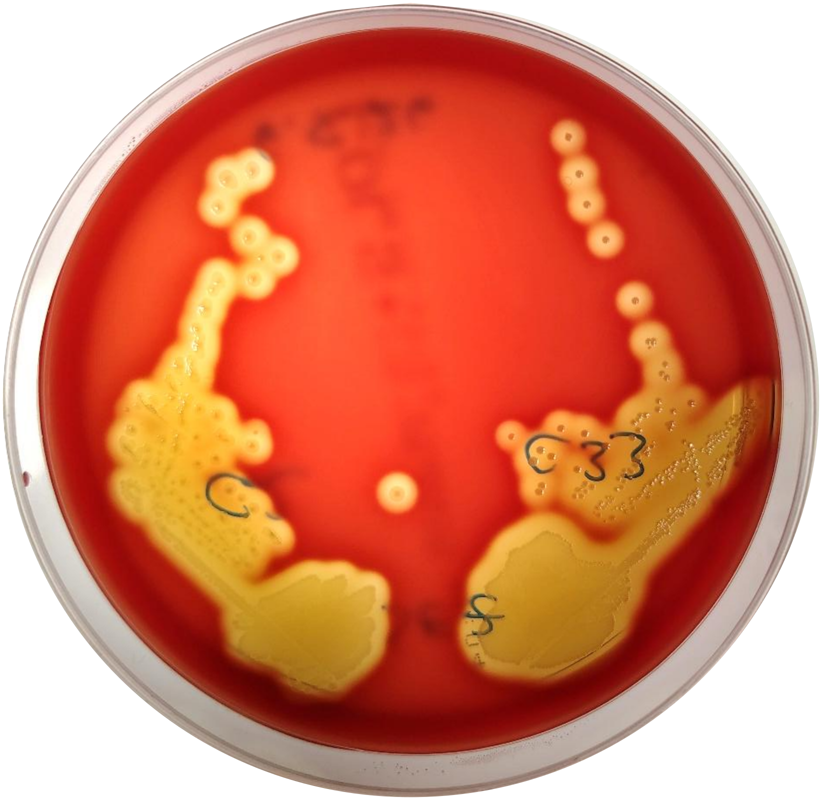


**Suppl. Fig. S2** Blood agar plate showing cultures of strain C2 (left) and C33 (right) after 24h incubation at 37°C.

**Suppl. Table 1:** MLST results of SEZ strains C2 and C33.

| | ST | arcC | nrdE | proS | spi | tdk | tpi | yqiL |
|---|---|---|---|---|---|---|---|---|
| **C2** | **26** | 15 | 13 | 7 | 18 | 11 | 14 | 24 |
| **C33** | **212** | 10 | 14 | 22 | 14 | 15 | 10 | 33 |

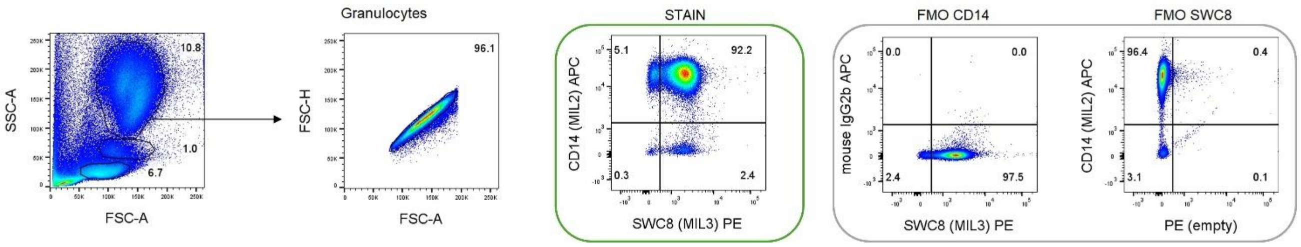


**Suppl. Fig. S3: Leukocytes** were labelled with anti-CD14 (Biorad MCA1218GA), Mouse IgG2b isotype control (Biolegend, 400301), anti-SWC8 (Biorad MCA1219), and secondary antibodies (APC anti-mouse IgG2b (Biolegend RMG2b-1) and PE anti-mouse IgM (Miltenyi 130-095-908)). Granulocytes were gated using SSC and FSC, and the granulocyte gate was investigated in APC and PE channels. More than 90% of the cells in the estimated gate were double-positive for the markers CD14 and SWC8, as expected for neutrophils(37). CD14 negative and SWC8 positive cells in the granulocyte gate can account for eosinophils and double-negative cells for basophils. The 5% CD14 positive, SWC8 negative cells may be monocytes gated along with granulocytes.

**Suppl. Table 2: Control of FarRed and CFSE labelling of SEZ strains by Flow Cytometry measurement with BD LSRFortessa.**

**A** **FarRed** labelled SEZ out of all SEZ

| | C2 | C2Δ*szm* | C33 | C33Δ*szm* |
|---|---|---|---|---|
| | 99,00% | 99,90% | 97,90% | 99,90% |
| | 99,50% | 99,80% | 99,40% | 99,90% |
| | 99,70% | 99,90% | 98,50% | 99,90% |
| mean | 99,40% | 99,87% | 98,60% | 99,90% |

**B** **CFSE** labelled SEZ out of all SEZ

| | C2 | C2Δ*szm* | C33 | C33Δ*szm* |
|---|---|---|---|---|
| | 95,60% | 99,40% | 89,70% | 99,20% |
| | 97,40% | 99,70% | 95,20% | 99,20% |
| | 98,60% | 99,70% | 94,10% | 99,50% |
| mean | 97,20% | 99,60% | 93,00% | 99,30% |

## Suppl. Table 3: Score sheet for clinical signs in animal experiment with SEZ infection

| parameter | score | | | | | | | |
|---|---|---|---|---|---|---|---|---|
| | 0 | 1 | 2 | 3 | 4 | 8 | 10 | 25 |
| body temperature | < 40°C | 40.0 - 40.2°C | 40.3 - 40.5°C | > 40.5°C | | | | |
| feed intake | good | moderate | | ceased | | | | |
| lameness | no | low-grade | | high-grade | | | | recumbency, polyarthritis |
| behaviour | fresh | | faint | | | | listless | central nervous system dysorder[a] |
| pose | typical | | kyphosis | vomitus | localized tremor | tremor in multiple body regions | | |
| respiratory signs | costo-abdominal breathing | | forced abdominal breathing | | | cyanosis (ears) | | Mouth breathing with foaming at the mouth and/or cyanosis |

[a] tetanic spasm, opisthotonus, convulsions

## Suppl. Table 4: Clinical Scores of piglets post intravenous SEZ infection (highest score out of three screening time points during the respective day). Upper 6 piglets were infected with SEZ C33, lower 6 were inoculated with PBS.

| | **0 dpi** | **1 dpi** | **2 dpi** | **3 dpi** | **4 dpi** | **5 dpi** | **6 dpi** | **7 dpi** | **8 dpi** | **9 dpi** | **10 dpi** | **11 dpi** | **12 dpi** | **13 dpi** | **14 dpi** |
|---|---|---|---|---|---|---|---|---|---|---|---|---|---|---|---|
| **857** | **2** | 0 | 0 | 0 | **2** | 0 | 0 | 0 | 0 | 0 | 0 | 0 | 0 | 0 | 0 |
| **890** | **3** | **5** | 0 | 0 | 0 | **1** | **2** | 0 | **1** | 0 | 0 | 0 | 0 | 0 | 0 |
| **716** | **1** | **8** | 0 | 0 | 0 | 0 | 0 | 0 | 0 | 0 | 0 | 0 | 0 | 0 | 0 |
| **694** | **3** | **6** | **2** | **3** | 0 | **2** | 0 | 0 | 0 | 0 | 0 | 0 | 0 | 0 | 0 |
| **661** | 0 | **6** | **1** | 0 | 0 | 1 | 0 | 0 | 0 | 0 | 0 | 0 | 0 | 0 | 0 |
| **684** | **4** | **3** | **4** | 0 | **3** | **6** | **4** | **2** | 0 | 0 | 0 | 0 | **25** | | |
| **16** | 0 | **2** | 0 | 0 | 0 | 0 | 0 | 0 | 0 | 0 | 0 | 0 | 0 | 0 | 0 |
| **806** | 0 | 0 | 0 | 0 | 0 | 0 | 0 | 0 | **7** | 0 | 0 | 0 | 0 | 0 | 0 |
| **896** | 0 | 0 | 0 | **1** | 0 | 0 | 0 | 0 | 0 | 0 | 0 | 0 | 0 | 0 | 0 |
| **875** | 0 | 0 | 0 | 0 | 0 | 0 | 0 | 0 | 0 | 0 | 0 | 0 | 0 | 0 | 0 |
| **678** | 0 | 0 | 0 | 0 | 0 | 0 | 0 | 0 | 0 | 0 | 0 | 0 | 0 | 0 | 0 |
| **874** | 0 | 0 | 0 | 0 | 0 | 0 | 0 | 0 | 0 | 0 | **1** | 0 | 0 | 0 | 0 |

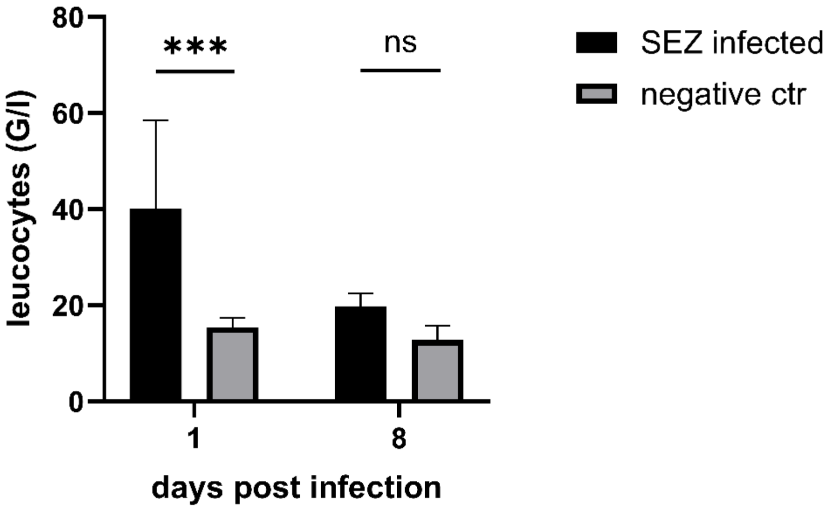


**Suppl. Fig. S4: Blood was collected in EDTA tubes for total and differential leukocytes count** using an automatic hematology analyzer (ADVIA 120, Siemens Healthcare Diagnostic Products GmbH, Marburg, Germany).

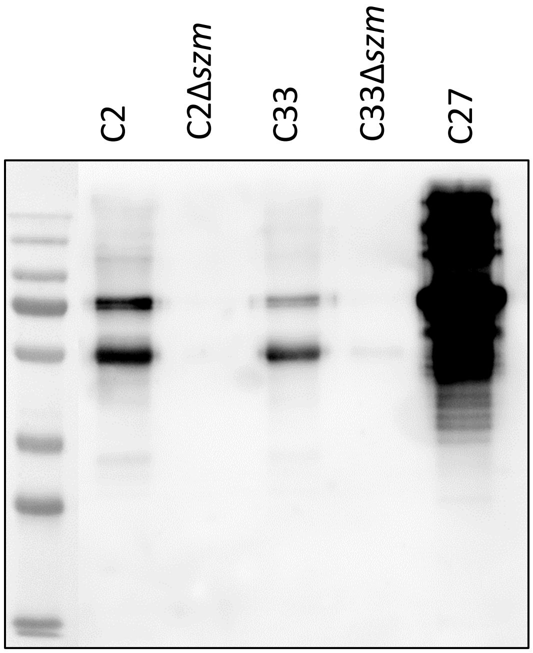


**Suppl. Fig. S5 Western Blot analysis of porcine fibrinogen bound to SEZ**

SEZ cells grown to mid-log phase and subsequently incubated with porcine plasma were analyzed using reducing SDS-PAGE. Western blot analysis was performed using rabbit anti-pig fibrinogen (ABIN491121, antibodies-online) and goat anti-rabbit IgG HRP (111-035-008, Dianova) antibodies.
